# ﻿*Thoreabaiyunensis* sp. nov. (Thoreales, Rhodophyta) and *T.okadae*, a new record from China

**DOI:** 10.3897/phytokeys.193.79667

**Published:** 2022-04-01

**Authors:** Jinfen Han, Fangru Nan, Jia Feng, Junping Lv, Qi Liu, Xudong Liu, Shulian Xie

**Affiliations:** 1 School of Life Science, Shanxi Key Laboratory for Research and Development of Regional Plants,Shanxi University, Taiyuan 030006, China Shanxi University Taiyuan China

**Keywords:** COI-5P, freshwater Rhodophyta, new taxon, phylogeny, *rbc*L, *Thorea*

## Abstract

The freshwater red algal order Thoreales has a triphasic life history, of which the “Chantransia” phase is a small filamentous sporophyte. The “Chantransia” stage is difficult to distinguish from species in the genus *Audouinella* by its morphological characteristics. In this study, five “Chantransia” isolates (GX41, GX81, GD224, GD225, GD228) were collected from Guangxi Zhuang Autonomous Region and Guangdong Province in China. Based on morphological data, all five isolates were similar to *A.pygmaea*, whereas sequence data from the large subunit of ribulose-1,5-bisphosphate carboxylase/oxygenase (*rbc*L) gene and the 5’ region of the mitochondrial cytochrome oxidase I gene (COI-5P) determined that these specimens represented the “Chantransia” stage of two species in the genus *Thorea* rather than *Audouinella*. Phylogenetic analyses of the concatenated genes supported the proposal of a new species, *T.baiyunensis*, and a new geographic record of *T.okadae*, a species previously described only in Japan. Therefore, combined with previous records, four species of this genus are now recognized in China, including *T.hispida*, *T.violacea*, *T.baiyunensis* and *T.okadae*.

## ﻿Introduction

Currently, more than 7,000 red algal species are reported worldwide, of which freshwater species only account for 3%. In the Rhodophyta, only four orders (Balbianiales, Batrachospermales, Compsopogonales, and Thoreales) have all members that are strictly freshwater species. Among the freshwater red algae, the genus *Thorea* Bory was established by Bory de Saint-Vincent (1808), since when its taxonomic status has undergone numerous changes. Based on the pit plug structure, Pueschel and Cole (1982) removed the Thoreaceae and Batrachospermaceae from the Nemaliales and established the order Batrachospermales. However, molecular research showed that species in the Thoreaceae were distantly related to the other Batrachospermales taxa (Vis et al. 1998). Subsequently, based on DNA sequence data, secondary structure of the SSU gene and characteristics of the outer layer of the pit plug, Müller et al. (2002) established the Thoreales. Species of this order are characterized by having multiaxial gametophytes, a uniaxial “Chantransia” stage, and pit plugs with two cap layers, the outer one of which is usually plate-like.

As currently recognized, the Thoreales contain a single family Thoreaceae with two genera, *Thorea* and *Nemalionopsis*. The main difference between the genera *Thorea* and *Nemalionopsis* is that *Thorea* has assimilatory filaments that are not contained in a common gelatinous matrix with reproductive structures (carpogonia, spermatangia, carposporangia and monosporangia) at their base and a lower ratio of sporangial branch to assimilatory filament length and loose aggregation (Skuja 1934; Sheath and Cole 1993). In a recent review of *Thorea*, sixteen species were recognized worldwide (Johnston et al. 2018). However, only two species (*T.hispida* (Thore) Desvaux, *T.violacea* Bory) of this genus have been reported in China (Shi 2006). The type species of the genus, *T.hispida*, was widely distributed in China, while *T.violacea* was only reported in Guizhou Province (Xie and Shi 2003; Shi 2006). Asexual reproduction by monosporangia is commonly reported in *Thorea*, while sexual reproduction is observed in a few species, including *T.hispida*, *T.conturba* Entwisle & Foard, *T.okadae* Yamada, *T.bachmannii* C.Pujals ex R.G.Sheath, M.L.Vis & K.M.Cole, *T.kokosinga-pueschelii* E.T.Johnston & M.L.Vis, and *T.mauitukitukii* E.T.Johnston, K.R.Dixon, J.A.West & M.L.Vis (Kumano 2002; Johnston et al. 2018). Among them, *T.okadae* is widely distributed in Japan (Yamada 1949; Johnston et al. 2018), but so far, this species has not been reported in other countries and regions. In addition, according to Kumano (2002), *T.okadae* is the largest species in the genus *Thorea*, and the length of its gametophyte often exceeds 1 m, and may reach 3 m.

Like other sexually reproductive species of freshwater red algae, *Thorea* species have a triphasic life history, including gametophyte, carposporophyte, and a diminutive diploid sporophyte termed “Chantransia” stage. Recently, several studies have focused on the phylogenetic affinities of the “Chantransia” stages of *Thorea* and the relationship between the isolates in this stage and a phylogenetically distant genus *Audouinella* Bory (Necchi and Oliveira 2011; Han et al. 2021). Based on these studies, new species were proposed, new distributions were found, and higher *Thorea* species diversity was revealed (Johnston et al. 2018; Han et al. 2021). In terms of morphological characters, the “Chantransia” stages of the Thoreales are very similar to those of *Audouinella* taxa, although some studies have indicated that thallus color can be used as a reliable character (Zucchi and Necchi 2003) to distinguish true *Audouinella* (reddish) from “Chantransia” (bluish). However, “Chantransia” stages of the Thoreales can be brownish (Chiasson et al. 2007), and some species of *Sheathia* can be brownish to reddish in addition to bluish (Han et al. 2020). Thus, morphological characteristics that can unequivocally distinguish them have not been found (Pueschel et al. 2000; Chiasson et al. 2005). It was not until the emergence of molecular data that the “Chantransia” stage could be used for species identification. Recently, researchers have shown that some “Chantransia” isolates of *Thorea* were misclassified as *Audouinella* (Han 2012; Chen et al. 2014; Nan et al. 2016). Based on different gene markers, Han (2012), Chen et al. (2014) and Nan et al. (2016) proposed that *A.sinensis* C.-C.Jao and *A.heterospora* S.L.Xie & Y.J.Ling were “Chantransia” of *T.hispida* rather than belong to *Audouinella*. Besides, numerous surveys (Pueschel et al. 2000; Zucchi and Necchi 2003; Chiasson et al. 2005, 2007; Necchi and Oliveira 2011; Han et al. 2020) demonstrated that eleven species, including members of the Thoreales (*T.hispida* and *N.tortuosa* Yoneda & Yagi), were associated with “*A.pygmaea*”. Furthermore, the molecular phylogeny based on the *rbc*L, COI-5P and the plastid 23S rRNA (UPA) genes unequivocally demonstrated that a sample morphologically similar to *A.macrospora* (Wood) Sheath & Burkholder represented the “Chantransia” phase of *T.hispida* (Han et al. 2021).

It is therefore evident that the morphological difference between “Chantransia” stages and *Audouinella* is not clear. Relying solely on traditional morphological methods can often cause confusion in the classification and identification of *Audouinella*-like freshwater red algae. All samples in this study are “Chantransia” stages, meaning that key diagnostic morphological characters were unavailable. Therefore, the present work has attempted to use two molecular markers (*rbc*L and COI-5P) to infer the phylogenetic position of all isolates in this study. In addition, a secondary aim of this investigation is to provide reference for local resource survey of freshwater red algae biodiversity in China.

## ﻿Methods

**Figure 1.**
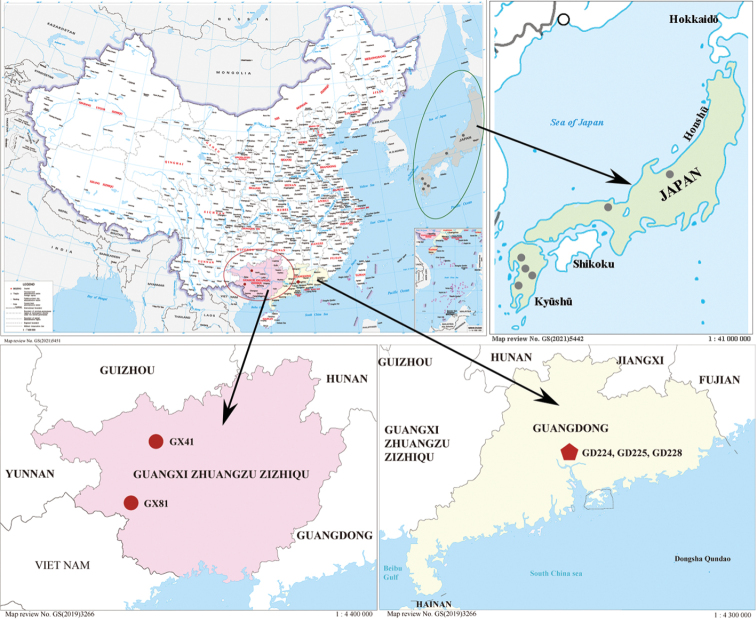
Map showing approximate locations of samples investigated in this study. More detailed location information is provided in Table 1. The red circles and the green circles indicate the locations of *Thoreaokadae* in China and Japan, respectively.

Five “Chantransia” specimens (GX41, GX81, GD224, GD225 and GD228) were collected from Guangxi Zhuang Autonomous Region and Guangdong Province in China (Fig. 1, Table 1). Handheld meters (YSI Professional Plus Multiparameter Water Quality Instrument 19E102487, YSI Incorporated, Brannum Lane Yellow Springs, Ohio, USA) were used to measure water quality parameters, including water temperature and pH. Materials were picked up carefully using a knife and tweezers and transferred to the laboratory as soon as possible. After transfer to the laboratory, the samples were rinsed with sterile water to remove impurities. Other algae attached to the samples were carefully removed using tweezers and other tools. Then microscopic examination was undertaken to ensure that all epiphytic algae were removed. Morphological and genetic analysis was performed for each sample. For observations and measurements, we used a BX-51 Olympus microscope equipped with a charge-coupled device (DP72; Olympus, Tokyo, Japan).

**Table 1.** Collection information and sequence accession numbers for taxa analyzed in this study.

Isolate	Locality with longitude and latitude	Collection date	Collector	Voucher number
GX41	Baimo Cave, Bama County, Guangxi Zhuang Autonomous Region, China (24°18.03'N, 107°05.96'E)	22 December 2019	Kunpeng Fang	GX19041
GX81	Tongling Great Falls, Jinxi County, Guangxi Zhuang Autonomous Region, China (23°43.10'N, 106°39.77'E)	23 December 2019	Kunpeng Fang	GX19081
GD224	Baiyun Mountain, Guangzhou, Guangdong province, China (23°36.00'N, 113°49.53'E)	22 November 2020	Jinfen Han and Kunpeng Fang	GD20224
GD225	Baiyun Mountain, Guangzhou, Guangdong province, China (23°36.00'N, 113°49.53'E)	22 November 2020	Jinfen Han and Kunpeng Fang	GD20225
GD228	Baiyun Mountain, Guangzhou, Guangdong province, China (23°36.00'N, 113°49.53'E)	22 November 2020	Jinfen Han and Kunpeng Fang	GD20228

Total DNA was extracted following the protocol originally described by Saunders (1993) and revised by Vis and Sheath (1997). Two molecular markers, *rbc*L and COI-5P, were amplified using the primers and protocols described by Vis et al. (1998) and Saunders (2005). The PCR products with their amplification primers were sent to BGI Tech Corporation (Beijing, China) for sequencing on an ABI 3730XL sequencer. Sequences generated in this study were submitted to the GenBank databases (Table 2). Additional related sequence data of the Thoreales order and outgroup taxa *Batrachospermum* were downloaded from GenBank (http://www.ncbi.nlm.nih.gov/) (Suppl. material 3: Table S1). The 58 *rbc*L, and 47 COI-5P sequences were aligned by CLUSTAL-X 2.0 (Thompson et al. 1997) and MEGA 5.0 (Tamura et al. 2011). Pairwise distance and the number of nucleotide variances for the taxa’s molecular markers were calculated in MEGA 5.0. For phylogenetic analyses, the appropriate models for the sequence evolution were determined by MODELTEST 3.7 (Posada and Buckley 2004). The parameters for the concatenated sequences (*rbc*L and COI-5P) maximum likelihood (ML) analyses were as follows: GTR+I+G model; gamma distribution=0.5411; proportion of invariable sites=1.1344; base frequencies A=0.3352, C=0.1247, G=0.1641, and T=0.3760; and rate matrix A–C=3.7852, A–G=9.4223, A–T=1.1874, C–G=0.6077, and C–T=20.8378. PHYML software (Felsenstein 1981; Guindon and Gascuel 2003) was utilized to construct the ML trees with 1,000 replicates of bootstrap analysis. Bayesian Inferences (B.I.) were performed in MRBAYES VERSION 3.1.2 (Ronquist and Huelsenbeck 2003) and runs 5,000,000 generations sampling every 1,000 generations until the standard error was lower than 0.01. The resulting phylogenetic trees were edited using FIGTREE 1.3.1 (http://tree.bio.ed.ac.uk/software/figtree/).

**Table 2.** GenBank accession numbers of *rbc*L and COI-5P sequences generated in this study.

Taxon	Isolate	rbcL accession No.	COI-5P accession No.
*Thoreaokadae*	GX41	MZ648088	MZ676778
*T.okadae*	GX81	MZ648089	MZ676779
*T.baiyunensis*	GD224	MZ648090	MZ676780
*T.baiyunensis*	GD225	MZ648091	MZ676781
*T.baiyunensis*	GD228	MZ648092	MZ676782

## ﻿Results

### ﻿Molecular analysis

The *rbc*L data matrix included 56 specimens of Thoreales and 2 outgroup taxa, consisting of 1203 characters, of which 410 (34.08%) were variable and 380 (31.59%) were parsimony informative. The *rbc*L *p*-distance among the five “Chantransia” isolates and other specimens of order Thoreales (Suppl. material 4: Table S2) showed that divergences between the isolates (GX41 and GX81) collected from Guangxi Zhuang Autonomous Region and five *T.okadae* specimens previous reported from Japan were 0%–1%, corresponding to 4–6-bp differences, the average distance between these two samples (GX41 and GX81) and the other species of genus *Thorea* was 8.5%. These results supported the close relationship between the two isolates (GX41 and GX81) and *T.okadae* and further substantiated that GX41 and GX81 were the “Chantransia” of *T.okadae*. However, three other isolates (GD224, GD225, and GD228) collected from Guangdong Province had unique sequences. The distances between them and the other species of *Thorea* were higher than the intraspecific distances of this genus (10.88% VS 8.28%).

The COI-5P sequence determined for the five “Chantransia” isolates from this study were 676 bp, of which 297 (43.93%) were variable and 273 (40.38%) were parsimony informative. The pairwise distance among the “Chantransia” isolates collected in this study and other Thoreales taxa (Suppl. material 5: Table S3) showed that isolates GX41 and GX81 were closely related to *T.okadae* from Japan. The mean *p*-distance between samples from China and Japan was 3%. The intraspecific *p*-distances of species in this genus were 0–6%, whereas the interspecies *p*-distances of COI-5P sequences among the species of *Thorea* were 7.3–18.0%. For the remaining three isolates GD224, GD225, and GD228, their pairwise distances with other Thoreales specimens also supported the unique taxonomic status of GD224, GD225 and GD228: the *p*-distance between them and the other species of *Thorea* was 14.17%, which was larger than the intraspecific distance of this genus (0–6%).

**Figure 2.**
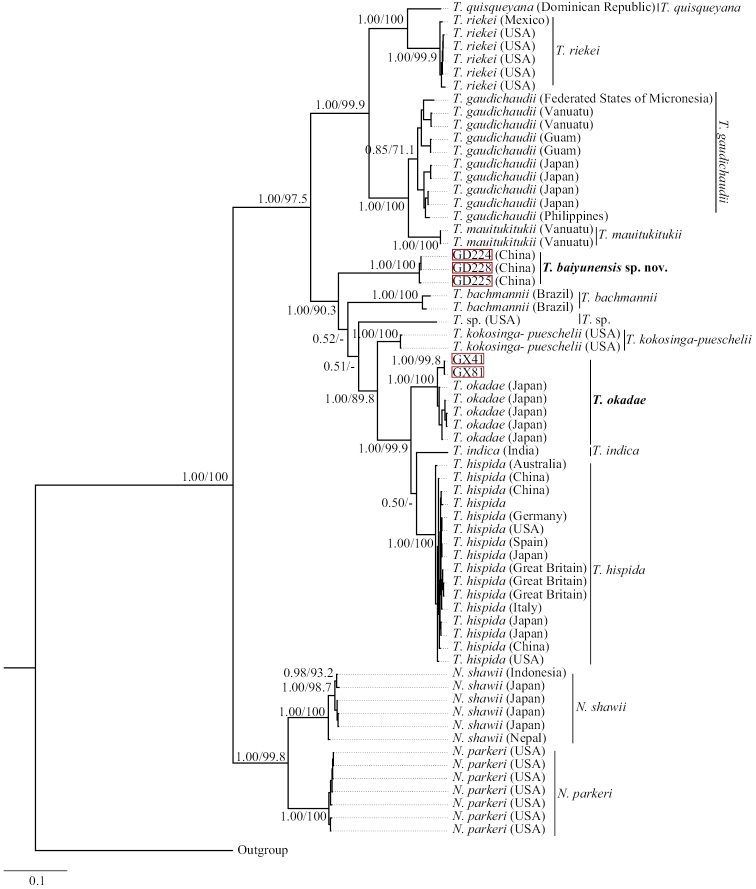
Bayesian inference tree for Thorea and Nemalionopsis based on concatenated sequences from *rbc*L and COI-5P genes. Support values for all analyses are shown as follows: Bayesian posterior probabilities/ML bootstrap. ‘-’ denotes <50% support for those analyses at that node. All new sequences generated in this study are indicated in red boxes.

Phylogenetic analyses based on single gene and concatenated genes produced trees with similar tree topologies, such that only the concatenated B.I. tree with supporting values calculated from two methods was displayed (Fig. 2). The single gene phylogenetic trees based on *rbc*L and COI-5P are shown in the supporting materials (Suppl. material 1, 2: Figs S1, S2).

The phylogenetic analyses strongly supported the monophyly of the Thoreales, *Nemalionopsis* and *Thorea* (Fig. 2). The *Thorea* clade contained two major subclades, one comprising four species: *T.quisqueyana* E.T.Johnston & M.L.Vis, *T.riekei* Bischoff, *T.gaudichaudii* C.Agardh, and *T.mauitukitukii* (1.00/99.9); and the second subclade including six species (*T.baiyunensis*, *T.bachmannii*, *T.kokosinga-pueschelii*, *T.okadae*, *T.indica* Necchi, E.K.Ganesan & J.A.West, and *T.hispida*) and an undetermined species (1.00/90.3). Isolates from this study were in the second subclade and formed two distant clusters. The isolates GX41 and GX81 were within a clade with five *T.okadae* specimens collected from Japan. This relationship was well supported by Bayesian posterior probabilities (100%) and ML bootstrap (100%). The remaining three isolates, GD224, GD225, and GD228 were an independent clade distantly related from any of the previously described species with high support values (1.00/90.3).

### ﻿Morphological observations

**Figure 3.**
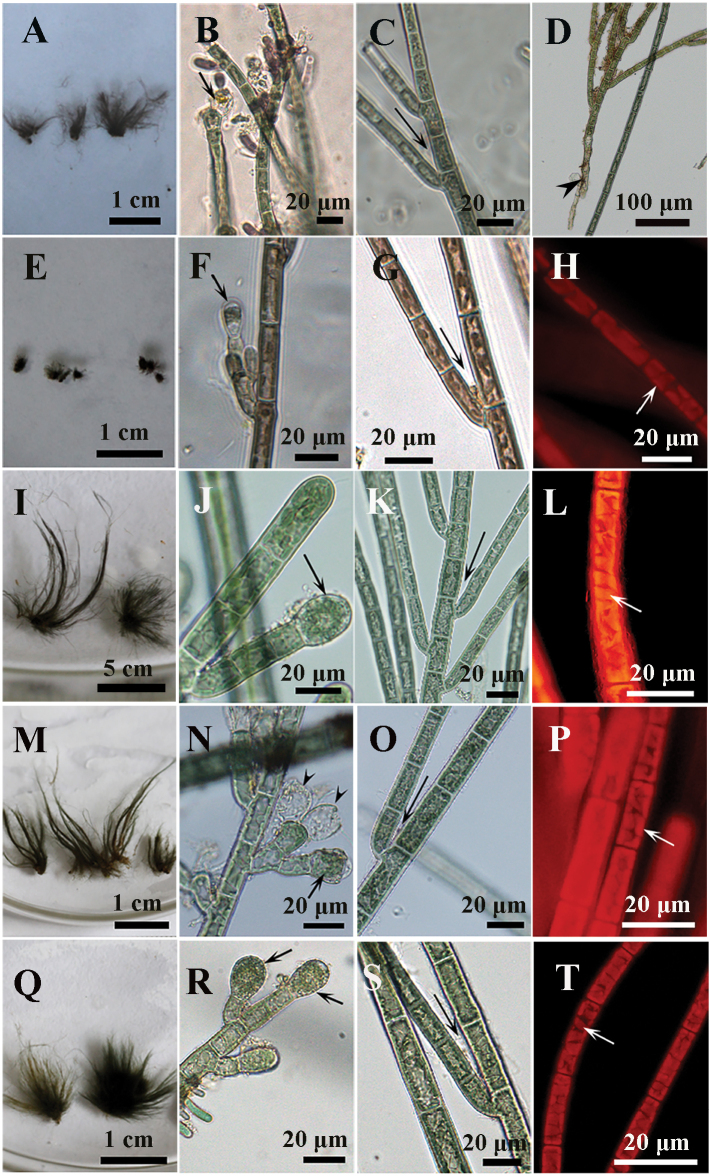
Morphological characters of samples investigated in this study **A–D** sample GX41 **A** morphological observation of the tufts of erect filament, bluish in color **B** monosporangial branch with ovoid monosporangium (black arrow) **C** filament showing branch angle ≤ 25° (black arrow) **D** erect filaments arise from basal system consisting of a prostrate mass with sparse rhizoids (black arrowhead) **E–H** sample GX81 **E** morphological observation of the tufts of erect filament, brownish in color **F** monosporangial branch with ovoid monosporangium (black arrow) **G** filament showing branch angle < 25° (black arrow) **H** cells have parietal laminate or irregularly lobed Chloroplast (white arrow) **I–L** sample GD224 **I** morphological observation of the tufts of erect filament, bluish in color **J** monosporangial branch with obovoidal monosporangium (black arrow) **K** filament showing branch angle < 25° (black arrow) **L** cells have parietal laminate or irregularly lobed Chloroplast (white arrow) **M–O** sample GD225 **M** morphological observation of the tufts of erect filament, bluish in color **N** monosporangial branch with obovoidal monosporangium (black arrow) **O** filament showing branch angle < 25° (black arrow) **P** cells have parietal laminate or irregularly lobed Chloroplast (white arrow) **Q–T** sample GD228 **Q** morphological observation of the tufts of erect filament, bluish in color **R** monosporangial branch with obovoidal monosporangium (black arrow) **S** filament showing branch angle < 25° (black arrow) **T** cells have parietal laminate or irregularly lobed Chloroplast (white arrow).

The morphometric data showed that all samples used in this study fit the morphological description of *Audouinellapygmaea* (Roth) Duby, although the tuft length of GX81 is significantly smaller than others (Fig. 3A–T, Table 3). Their characteristics are as follows: tuft-shape, 1.2–25.9 mm length, bluish or brownish in color; basal portion consisting of an irregular prostrate system of densely aggregated filaments; erect filaments dense, irregular branched, apical cells obtuse, without hair; vegetative cells of main branches cylindrical, 8.2–68.2 μm in length and 7.2–22.7 μm in diameter; monosporangial branches short, mostly grow at the tip of vegetative branch, with few small branches; monosporangia are obovoidal, 11.8–22.7 µm in length and 7.2–17.3 µm in diameter.

**Table 3.** Morphological characteristics of specimens in this study.

Thalli characteristics	GX41	GX81	GD224	GD225	GD228
Color	Bluish	Brownish	Bluish	Bluish	Bluish
Height (mm)	(4.8) 5–9.4 (10.0)	(1.2) 1.5–3.5 (4.4)	(3.4) 4.1–9.3 (10.8)	(4.1) 5.4–22.9 (25.9)	(8.5) 8.8–12.9 (13.6)
Branch angle*	≤ 25°	< 25°	< 25°	< 25°	< 25°
Vegetative cells Length (μm)	(8.2) 10–28.2 (31.8)	(20.9) 22.7–54.5 (59.1)	(12.7) 13.6–63.6 (68.2)	(21.8) 22.7–59.1(60.9)	(22.7) 23.6–43.6 (45.5)
Vegetative cells Diameter (μm)	(7.2) 8.2–10.9 (11.8)	(8.2) 9.1–10.9 (11.8)	(8.2) 10.9–15.5 (16.4)	(10.9) 11.8–15.5 (16.4)	(11.8) 12.7–20.9 (22.7)
Monosporangia Shape	Obovoidal	Obovoidal	Obovoidal	Obovoidal	Obovoidal
Monosporangia Length (μm)	(12.7) 13.6–14.5 (15.5)	(12.7) 13.6–18.2 (19.1)	(11.8) 12.7–21.8 (22.7)	(12.7) 13.6–17.3 (19.1)	(13.6) 14.5–21.8 (23.6)
Monosporangia Diameter (μm)	(7.2) 8.2–14.5 (15.5)	(8.2) 9.1–12.7 (13.6)	(10.9) 11.8–15.5 (16.4)	(10.9) 11.8–13.6 (14.5)	(10.9) 11.8–16.4 (17.3)
Chloroplast number	2–4	2–4	2–4	2–4	2–4
Chloroplast shape	laminate or irregularly lobed	laminate or irregularly lobed	laminate or irregularly lobed	laminate or irregularly lobed	laminate or irregularly lobed

* Definition follows Necchi and Zucchi (1995).

### ﻿Taxonomic proposals

The genetic distance and phylogenetic analysis based on *rbc*L, COI-5P and the concatenated genes all supported the identification of new species described below.

#### ﻿ Thorea baiyunensis

Taxon classificationPlantaeThorealesThoreaceae

Han, Nan & Xie sp. nov.

6B4AE1DE-D855-5B71-9170-499BF5CF4286

Fig. 3I–T

##### Description.

Known only from the “Chantransia” sporophyte generation. Plant macroscopic, up to 25.9 mm, bluish; basal portion consisting of an irregular prostrate system of densely aggregated filaments; lateral branches developing at angle < 25°. Vegetative cells of main branches cylindrical, (12.7) 13.6–63.6 (68.2) μm long and (8.2) 10.9–20.9 (22.7) μm in diameter. Monosporangia numerous, mostly grow at the tip of 1–5-celled, short branchlets, singly or in clusters, obovoidal, (11.8) 12.7–21.8 (23.6) μm long and (10.9) 11.8–16.4 (17.3) μm in diameter. Chloroplasts laminate or irregularly lobed, 2–4 per cell.

##### Diagnosis.

Diagnostic DNA sequence: *rbc*L and COI-5P (accession number: MZ648090, MZ648091, and MZ648092 for *rbc*L and MZ676780, MZ676781, and MZ676782 for COI-5P).

##### Type.

China, Guangdong Province, Baiyun Mountain, 540 m alt., 23°36.00'N, 113°49.53'E: epilithic on the rocks in spring water, November 2020, J.F. Han & K.P Fang (Holotype: SXU-SAS18040; Paratype: SXU-SAS18041). Deposited in Herbarium of Shanxi University (SXU), Shanxi University, Taiyuan, Shanxi Province, China.

##### Habitat and distribution.

**Figure 4.**
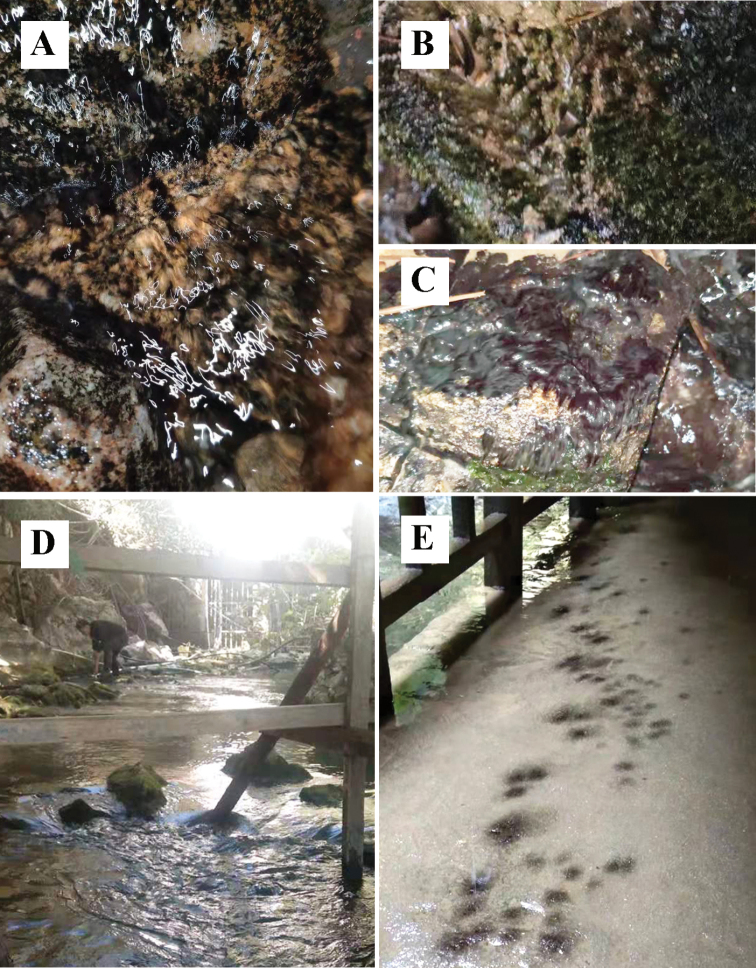
Habitat of *Thoreabaiyunensis* and *T.okadae***A–C** habitat of *Thoreabaiyunensis***D, E** habitat of *T.okadae*.

Baiyun Mountain (23°36.00'N, 113°49.53'E), Guangdong Province, China: on the rocks in spring water; water temperature 17.3–20.6 °C, pH 6.5–7.6 (Figs 1, 4A–C).

##### Etymology.

The species epithet refers to the type locality (Baiyun Mountain, China).

##### Authentic strain.

SXU-GD20224.

## ﻿Discussion

Several studies have confirmed that it is difficult to distinguish “Chantransia” stages from true *Audouinella* based only on morphological characteristics, which often hinders species identification and brings confusion to the classification system of freshwater red algae (Chiasson et al. 2005; Necchi and Oliveira 2011; Han et al. 2020). More surprising is that even individuals with completely different gametophyte morphology, belonging to different species, genera or orders, may also produce “Chantransia” stages with extremely similar morphology. In this study, morphological data showed that all samples collected from South China were similar and within the circumscription of *A.pygmaea*. However, molecular analysis confirmed that they were not *Audouinella* taxa but the “Chantransia” stages of two different *Thorea* species, *T.okadae* and *T.baiyunensis*. Based on sequence data and culture studies (Pueschel et al. 2000; Zucchi and Necchi 2003; Chiasson et al. 2005, 2007; Necchi and Oliveira 2011; Han et al. 2020), eleven species of orders Batrachospermales and Thoreales have been confirmed to form “Chantransia” stages similar to *A.pygmaea* in morphology. Thus, at least thirteen species of the orders Batrachospermales and Thoreales are associated with “*A.pygmaea*” so far. It is clear that the morphological characteristics of the “Chantransia” stages cannot be used to determine the identity individual species (Necchi and Oliveira 2011; Han et al. 2021). However, how genetic information and environmental factors regulate the morphological expression of “Chantransia” stages remain unclear.

With the widespread application of molecular data in the taxonomy of freshwater red algae, the view that morphologically simple organisms have considerable genetic diversity and species richness has been widely recognized (Johnston et al. 2018; Han et al. 2020, 2021). In the past few years, several new species of the Batrachospermales and Thoreales were proposed based on the “Chantransia” sporophyte generation, such as *T.quisqueyana*, *Sheathiashimenxiaensis* J.-F.Han, F.-R.Nan et S.-L.Xie, *S.jiugongshanensis* J.-F.Han, F.-R.Nan et S.L.Xie, and *S.qinyuanensis* J.-F.Han, F.-R.Nan et S.L.Xie (Johnston et al. 2018; Han et al. 2020, 2021). In this article, based on the *rbc*L and COI-5P sequences, all five samples utilized in this study were unquestionably proved to be the “Chantransia” stages of two species of genus *Thorea*, whose gametophytes have never been reported in China before. Therefore, taking into account the two stages, the geographical distribution of genus *Thorea* would be larger, and the number of species would be more than that identified only by morphological data. Combined with previous reports in China, four species of genus *Thorea* have been recognized, including *T.hispida*, *T.violacea*, *T.baiyunensis*, and *T.okadae*. At the same time, the number of the *Thorea* distribution sites is proposed to have expanded to eight, including Shanxi, Jiangsu, Yunnan, Guizhou, Hunan, Henan, Guangdong provinces and Guangxi Zhuang Autonomous Region. This study reinforces the importance of collecting and sequencing specimens of “Chantransia” stages as a tool to reveal the hidden diversity of freshwater red algae of the orders Batrachospermales and Thoreales in different regions of the world.

In 1949, Yamada reported a new species, *T.okadae*, when studying the specimens of genus *Thorea* in Kagoshima prefecture, Japan (Yamada 1949). According to Kumano (2002) and Kozono (2020), *T.okadae* is the largest species known in the genus *Thorea* and is widely distributed in different areas of Honshu Island and Kyushu Island in Japan. This species has long been considered endemic to Japan. However, in this study we reported two “Chantransia” stages (GX41 and GX81) of *T.okadae* from Guangxi Zhuang Autonomous Region, China. Sequence analysis based on *rbc*L and COI-5P showed that there were only a few base differences between *T.okadae* from China and Japan. As shown in Fig. 1, the distribution of *T.okadae* in China (red circle) is far away from that in Japan (green circle). However, these regions all belong to the subtropical monsoon climate, which means that these places have similar climatic conditions, including light, temperature and precipitation. According to Higa et al. (2007), both gametophytes and “Chantransia” stage specimens of *T.okadae* were observed in Kikuchi River, Japan where water pH is 6.6–7.5, temperature is 26.3 °C in summer and 9.5 °C in winter. In addition, the mean day length of this place ranged from 9.9 h in December to 14.3 h in June. In this study, the “Chantransia” isolates of *T.okadae* were collected in Baimo Cave and Tongling Great Falls (Fig. 4D, E). The habitat characteristics of these two places are as follows: mean water temperature ranged from 14 °C to 21 °C and pH values fluctuated between 7.2 and 7.8, while the day length is about 10.7 h in December and 13.5 in June. The environmental factors of Baimo Cave and Tongling Great Falls were similar to those of Kikuchi River, thus, it is not surprising that *T.okadae* was found in Guangxi Zhuang Autonomous Region, China. It is therefore reasonable to infer that *T.okadae* also occurs in other areas of China with the same regional climate.

In a study on the seasonality of gametophyte occurrence, maturation and fertilization of the freshwater red alga *T.okadae*, Higa et al. (2007) pointed out that gametophytes generally form from December and can be observed from early autumn to late spring, while “Chantransia” stages (sporophytes) last throughout the year. The “Chantransia” stages of *T.okadae* in this study were collected in December 2019, but we did not find its gametophytes. We speculate that this may be due to the following reasons: firstly, since the samples were only collected in December, the gametophytes of *T.okadae* may have just begun to form. In this case, the small number of gametophytes and the short length of assimilatory filaments make them difficult to detect; additionally, the absence of the gametophytes of *T.okadae* may also be due to the environmental conditions that were not suitable for inducing their formation.

## Supplementary Material

XML Treatment for Thorea baiyunensis

## Supplementary materials

Supplementary material 1 Figure S1 Data type COL Explanation note Bayesian inference tree based on the rbcL gene sequences. Support values for all analyses are shown as follows: Bayesian posterior probabilities/ML bootstrap. ‘-’ denotes <50% support for that analysesat that node. All new sequences generated in this study are indicated in red boxes. https://binary.pensoft.net/file/667225This dataset is made available under the Open Database License (http://opendatacommons.org/licenses/odbl/1.0/). The Open Database License (ODbL) is a license agreement intended to allow users to freely share, modify, and use this Dataset while maintaining this same freedom for others, provided that the original source and author(s) are credited.Jinfen Han, Fangru Nan, Jia Feng, Junping Lv, Qi Liu, Xudong Liu, Shulian Xie

Supplementary material 2 Figure S2 Data type COL Explanation note Bayesian inference tree based on the COI-5P gene sequences. Support values for all analyses are shown as follows: Bayesian posterior probabilities/ML bootstrap. ‘-’ denotes <50% support for that analyses at that node. All new sequences generated in this study are indicated in red boxes. https://binary.pensoft.net/file/667226This dataset is made available under the Open Database License (http://opendatacommons.org/licenses/odbl/1.0/). The Open Database License (ODbL) is a license agreement intended to allow users to freely share, modify, and use this Dataset while maintaining this same freedom for others, provided that the original source and author(s) are credited.Jinfen Han, Fangru Nan, Jia Feng, Junping Lv, Qi Liu, Xudong Liu, Shulian Xie

Supplementary material 3 Table S1 Data type docx Explanation note Specimen information of sequences downloaded from the GenBank database. “–” denotes no related information for the specimen. https://binary.pensoft.net/file/667227This dataset is made available under the Open Database License (http://opendatacommons.org/licenses/odbl/1.0/). The Open Database License (ODbL) is a license agreement intended to allow users to freely share, modify, and use this Dataset while maintaining this same freedom for others, provided that the original source and author(s) are credited.Jinfen Han, Fangru Nan, Jia Feng, Junping Lv, Qi Liu, Xudong Liu, Shulian Xie

Supplementary material 4 Table S2 Data type xls Explanation note Pairwise distance (lower-left matrix) and number of nucleotide variance (upper-right matrix) of rbcL sequence among the taxa in this study. https://binary.pensoft.net/file/667228This dataset is made available under the Open Database License (http://opendatacommons.org/licenses/odbl/1.0/). The Open Database License (ODbL) is a license agreement intended to allow users to freely share, modify, and use this Dataset while maintaining this same freedom for others, provided that the original source and author(s) are credited.Jinfen Han, Fangru Nan, Jia Feng, Junping Lv, Qi Liu, Xudong Liu, Shulian Xie

Supplementary material 5 Table S3 Data type xls Explanation note Pairwise distance (lower-left matrix) and number of nucleotide variance (upper-right matrix) of COI-5P sequence among the taxa in this study. https://binary.pensoft.net/file/667229This dataset is made available under the Open Database License (http://opendatacommons.org/licenses/odbl/1.0/). The Open Database License (ODbL) is a license agreement intended to allow users to freely share, modify, and use this Dataset while maintaining this same freedom for others, provided that the original source and author(s) are credited.Jinfen Han, Fangru Nan, Jia Feng, Junping Lv, Qi Liu, Xudong Liu, Shulian Xie
