# Supplementary material for: ﻿Thoreabaiyunensis sp. nov. (Thoreales, Rhodophyta) and T.okadae, a new record from China
Source: PhytoKeys. 2022 Apr 1;193:107–23. doi: 10.3897/phytokeys.193.79667 (PMC9005493; doi:10.3897/phytokeys.193.79667)
Supplement: Supplementary material 1 — Figure S1 [file phytokeys-193-107-s001.pdf]

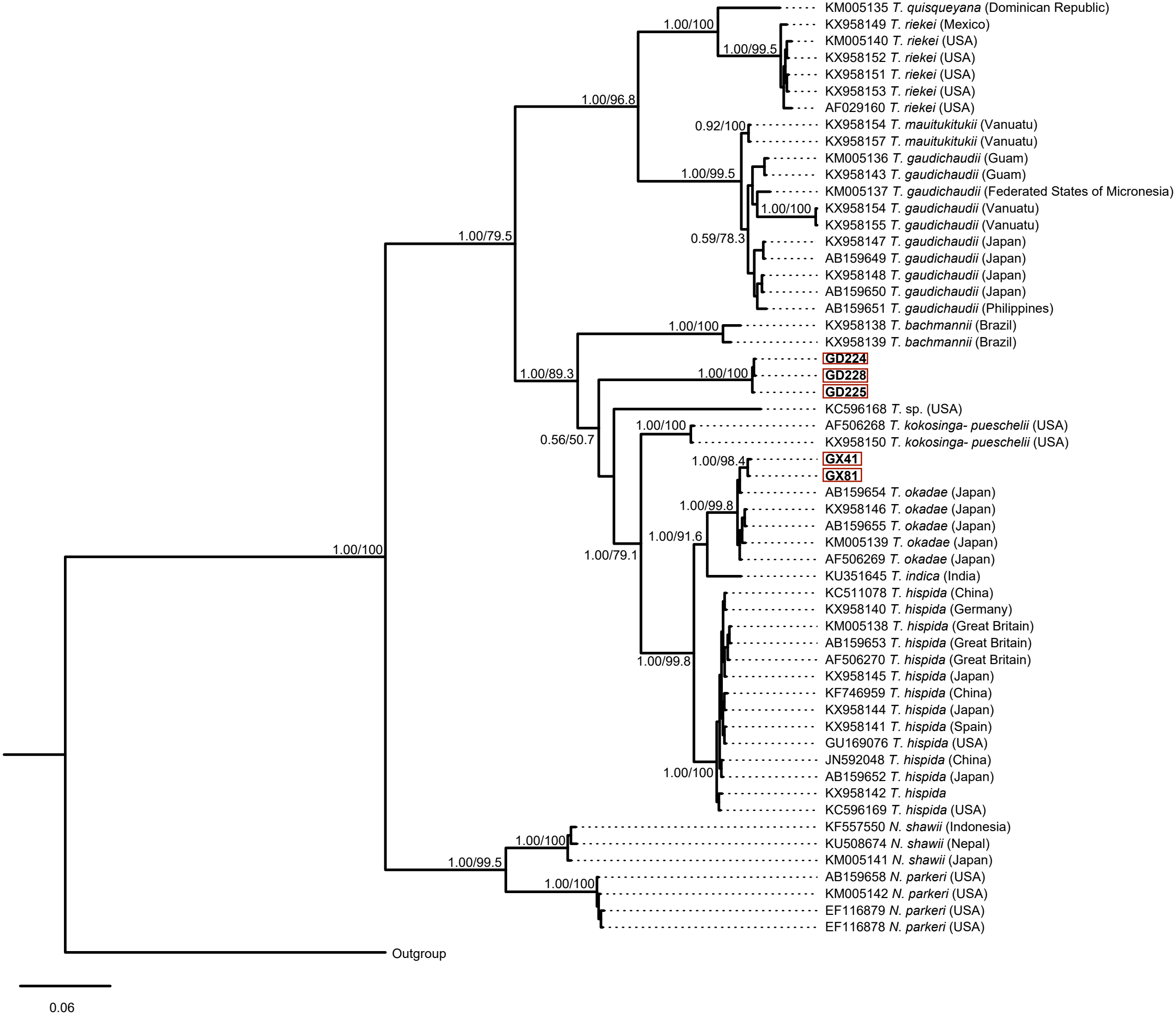

Figure S1. Bayesian inference tree based on the *rbc*L gene sequences. Support values for all analyses are shown as follows: Bayesian posterior probabilities/ML bootstrap. '-' denotes <50% support for that analysesat that node. All new sequences generated in this study are indicated in red boxes.
