# Supplementary material for: ﻿Thoreabaiyunensis sp. nov. (Thoreales, Rhodophyta) and T.okadae, a new record from China
Source: PhytoKeys. 2022 Apr 1;193:107–23. doi: 10.3897/phytokeys.193.79667 (PMC9005493; doi:10.3897/phytokeys.193.79667)
Supplement: Supplementary material 3 — Table S1 [file phytokeys-193-107-s003.docx]

***Thorea baiyunshanensis* sp. nov. (Thoreales, Rhodophyta) and *T. okadae* a new record from China**

Jinfen HAN^1^, Fangru NAN^1^, Jia FENG^1^, Junping LV^1^, Qi LIU^1^, Xudong LIU^1^, Shulian XIE^1^

1 *School of Life Science, Shanxi Key Laboratory for Research and Development of Regional Plants,Shanxi University, Taiyuan 030006, China*

Corresponding author: Shulian Xie ([xiesl@sxu.edu.cn](mailto:xiesl@sxu.edu.cn))

**Supplementary Tables**

Supplementary Table S1 Specimen information of sequences downloaded from the GenBank database. “−” denotes no related information for the specimen.

| Species | *rbc*L accession No. | COI-5P accession No. | Reference |
| --- | --- | --- | --- |
| *Batrachospermum gelatinosum* | GU810833 | GU810832 | House et al. 2010 |
| *B. gelatinosum* | GU810834 | GU810831 | House et al. 2010 |
| *Nemalionopsis* *parkeri* | − | KM055240 | unpublished |
| *N.* *parkeri* | EF116879 | − | Chiasson et al. 2007 |
| *N.* *parkeri* | EF116878 | − | Chiasson et al. 2007 |
| *N. parkeri* | − | KC596317 | Carlile and Sherwood 2013 |
| *N. parkeri* | − | KC596318 | Carlile and Sherwood 2013 |
| *N. parkeri* | − | KC596319 | Carlile and Sherwood 2013 |
| *N. parkeri* | KM005142 | KM055242 | unpublished |
| *N. shawii* | − | KX958097 | Johnston et al. 2018 |
| *N. shawii* | KF557550 | − | Johnston et al. 2014 |
| *N. shawii* | KM005141 | KM055241 | unpublished |
| *N. shawii* | − | KX958099 | Johnston et al. 2018 |
| *N. shawii* | DQ296122 | − | unpublished |
| *N.* *shawii* | − | KX958100 | Johnston et al. 2018 |
| *N. shawii* | − | KX958101 | Johnston et al. 2018 |
| *N. shawii* | KU508674 | KU508675 | Necchi et al. 2016 |
| *Thorea bachmannii* | KX958138 | KX958092 | Johnston et al. 2018 |
| *T. bachmannii* | KX958139 | − | Johnston et al. 2018 |
| *T. gaudichaudii* | KM005136 | KM055235 | unpublished |
| *T. gaudichaudii* | KX958143 | KX958096 | Johnston et al. 2018 |
| *T. gaudichaudii* | KM005137 | KM055236 | unpublished |
| *T. gaudichaudii* | KX958147 | KX958105 | Johnston et al. 2018 |
| *T. gaudichaudii* | AB159649 | − | unpublished |
| *T. gaudichaudii* | KX958148 | KX958106 | Johnston et al. 2018 |
| *T. gaudichaudii* | AB159650 | − | unpublished |
| *T. gaudichaudii* | AB159651 | − | unpublished |
| *T. gaudichaudii* | KX958154 | KX958114 | Johnston et al. 2018 |
| *T. gaudichaudii* | KX958157 | − | Johnston et al. 2018 |
| *T. hispida* | KC511078 | KC511076 | Ji et al. 2014 |
| *T. hispida* | JN592048 | − | unpublished |
| *T. hispida* | KF746959 | − | Feng et al. 2015 |
| *T. hispida* | KX958140 | KX958093 | Johnston et al. 2018 |
| *T. hispida* | KX958142 | KX958095 | Johnston et al. 2018 |
| *T. hispida* | KM005138 | KM055237 | unpublished |
| *T. hispida* | KC596169 | KC596320 | unpublished |
| *T. hispida* | − | KX958098 | Johnston et al. 2018 |
| *T. hispida* | KX958144 | KX958102 | Johnston et al. 2018 |
| *T. hispida* | KX958145 | KX958103 | Johnston et al. 2018 |
| *T. hispida* | KX958141 | KX958094 | Johnston et al. 2018 |
| *T. hispida* | AB159652 | − | unpublished |
| *T. hispida* | − | HM915870 | unpublished |
| *T. hispida* | − | KX958108 | Johnston et al. 2018 |
| *T. hispida* | GU169076 | − | unpublished |
| *T. indica* | KU351645 | KU351644 | Necchi et al. 2015 |
| *T. kokosinga- pueschelii* | KX958150 | − | Johnston et al. 2018 |
| *T. kokosinga- pueschelii* | AF506268 | − | Sheath et al. 2000 |
| *T. mauitukitukii* | KX958154 | KX958112 | Johnston et al. 2018 |
| *T. mauitukitukii* | KX958155 | KX958113 | Johnston et al. 2018 |
| *T. okadae* | KX958146 | KX958104 | Johnston et al. 2018 |
| *T. okadae* | AB159654 | − | unpublished |
| *T. okadae* | KM005139 | KM055238 | unpublished |
| *T. okadae* | AB159655 | − | unpublished |
| *T. quisqueyana* | KM005135 | KM055234 | unpublished |
| *T. riekei* | KX958149 | KX958107 | Johnston et al. 2018 |
| *T. riekei* | KM005140 | KM055239 | unpublished |
| *T. riekei* | AF029160 | − | Vis et al. 1998 |
| *T. riekei* | KX958151 | KX958109 | Johnston et al. 2018 |
| *T. riekei* | KX958152 | KX958110 | Johnston et al. 2018 |
| *T. riekei* | KX958153 | KX958111 | Johnston et al. 2018 |
| *T. riekei* | DQ523250 | − | Kapraun et al. 2007 |
| *Thorea sp.* | KC596168 | − | Carlile and Sherwood 2013 |

# References

Carlile AL, Sherwood AR (2013) Phylogenetic affinities and distribution of the Hawaiian freshwater red algae (Rhodophyta). Phycologia 52(3): 309–319. <https://doi.org/10.2216/12-097.1>

Chiasson WB, Johanson KG, Sherwood AR, Vis ML (2007) Phylogenetic affinities of the form taxon *Chantransia pygmaea* (Rhodophyta) specimens from the Hawaiian Islands. Phycologia 46(3): 257–262. https://doi.org/10.2216/06-79.1

Feng J, Chen L, Wang Y, Xie SL (2015) Molecular Systematics and Biogeography of *Thorea* (Thoreales, Rhodophyta) from Shanxi, China. Systematic Botany 40(2): 376–385. <https://doi.org/10.1600/036364415x688763>

House DL, Vandenbroek AM, Vis ML (2010) Intraspecific genetic variation of *Batrachospermum gelatinosum* (Batrachospermales, Rhodophyta) in eastern North America. Phycologia 49(5): 501–507. <https://doi.org/10.2216/09-104.1>

Ji L, Xie SL, Feng J, Chen L, Wang J (2014) Molecular systematics of four endemic Batrachospermaceae (Rhodophyta) species in China with multilocus data. Journal of Systematics and Evolution 52(1): 92–100. <https://doi.org/10.1111/jse.12058>

Johnston ET, Dixon KR, West JA, Buhari N, Vis ML (2018) Three gene phylogeny of the Thoreales (Rhodophyta) reveals high species diversity. Journal of phycology 54(2): 159–170. https://doi.org/10.1111/jpy.12618

Johnston ET, Lim PE, Buhari N, Keil EJ, Djawad MI, Vis ML (2014) Diversity of freshwater red algae (Rhodophyta) in Malaysia and Indonesia from morphological and molecular data. Phycologia 53(4): 329–341. <https://doi.org/10.2216/13-223.1>

Kapraun DF, Braly KS, Freshwater DW (2007) Nuclear DNA content variation in the freshwater red algal orders Batrachospermales and Thoreales (Florideophyceae, Nemaliophycidae). Phycologia 46(1): 54–62. <https://doi.org/10.2216/06-18.1>

Necchi OJr, Paiano MO, West JA, Ganesan EK, Goer SLD (2015) *Thorea indica* sp. nov.(Thoreales, Rhodophyta) from Uttar Pradesh, India. Algae 30(4): 265–274. <https://doi.org/10.4490/algae.2015.30.4.265>

Necchi OJr, West JA, Rai SK, Ganesan EK, Rossignolo NL, de Goër SL (2016) Phylogeny and morphology of the freshwater red alga *Nemalionopsis shawii* (Rhodophyta, Thoreales) from Nepal. Phycological Research 64(1): 11–18. <https://doi.org/10.1111/pre.12116>

Sheath RG, Mueller KM, Sherwood AR (2000) A proposal for a new red algal order, the Thoreales. Journal of Phycology 36(s3): 62. <https://doi.org/10.1046/j.1529-8817.1999.00001-185.x>

Vis ML, Saunders GW, Sheath RG, Dunse K, Entwisle TJ (1998) Phylogeny of the Batrachospermales (Rhodophyta) inferred from *rbc*L and 18S ribosomal DNA gene sequences. Journal of Phycology 34(2): 341–350. <https://doi.org/10.1046/j.1529-8817.1998.340341.x>
